# Adult Vitamin B12 Deficiency-Associated Pseudo-Thrombotic Microangiopathy: A Systematic Review of Case Reports

**DOI:** 10.7759/cureus.55784

**Published:** 2024-03-08

**Authors:** Venu M Ganipisetti, Babu Sriram Maringanti, Elvina C Lingas, Kushal Naha

**Affiliations:** 1 Hospital Medicine, Baystate Medical Center, Springfield, USA; 2 Hospital Medicine, University of New Mexico, Albuquerque, USA; 3 Hospital Medicine, Stony Brook University, Stony Brook, USA; 4 Hematology and Medical Oncology, University of Missouri-Columbia, Columbia, USA

**Keywords:** acquired ttp, vitamin b12 deficiency symptoms, plasmic score, pseudo-tma, pseudo-ttp, vitamin b12 deficiency anemia, cobalamin deficiency

## Abstract

Cobalamin-deficient thrombotic microangiopathy or vitamin B12 deficiency presenting as pseudo-thrombotic microangiopathy is a rare disorder that can be misdiagnosed as thrombotic thrombocytopenic purpura. Patients with this condition are at risk of receiving unnecessary plasmapheresis with a potential delay in appropriate therapy with vitamin B12 supplementation. There are no established diagnostic criteria for this condition in clinical practice. We performed a systematic review of case reports published between January 2018 and January 2023 to analyze the clinical characteristics, risk factors, and patterns of laboratory markers to improve the diagnostic criteria for this condition.

## Introduction and background

Vitamin B12 deficiency can present as pseudo-thrombotic microangiopathy (pseudo-TMA) that mimics thrombotic thrombocytopenic purpura (TTP) [1]. TTP is a life-threatening medical condition that results from a deficiency of ADAMTS13, a metalloproteinase enzyme that cleaves von Willebrand factor (vWF). ADAMTS13 deficiency can be acquired secondary to autoantibodies or can be congenital due to the inability to synthesize the enzyme. Regardless of the mechanism, loss of ADAMTS13 activity results in ultra-large vWF multimers that trigger platelet activation and aggregation, leading to microthrombi and tissue ischemia. When left untreated, this condition carries a mortality rate of over 90%. The cornerstone of management is plasma exchange, which simultaneously removes circulating autoantibodies and replenishes ADAMTS13 levels. Timely initiation of this life-saving therapy is critical and typically based on the clinical picture of hemolysis with schistocytosis and severe thrombocytopenia [2].

Cobalamin-deficient thrombotic microangiopathy presents with macrocytic anemia and ineffective erythropoiesis, leading to intramedullary hemolysis resulting in elevated lactate dehydrogenase (LDH), low haptoglobin, and schistocytes in the peripheral blood smear. Thrombocytopenia can also occur due to inadequate bone marrow production. This clinical combination of anemia with an elevated LDH, schistocytosis, and thrmobocytopenia closely mimics the presentation of TTP. Cobalamin-deficient thrombotic microangiopathy is a relatively rare disorder with a prevalence estimates as low as 0.6% to 2.5% of all vitamin B12 deficiency cases [3]. Due to its ambiguous presentation and comparative rarity, it is often misdiagnosed as TTP and treated inappropriately with plasmapheresis [4]. This is unfortunate, as plasmapheresis, while life-saving in appropriate circumstances, does carry a risk of complications. Moreover, although vitamin B12 supplementation is the mainstay of therapy, it is often delayed because of failure to recognize vitamin B12 deficiency as the underlying etiology [5]. Thus, early recognition and diagnosis of this condition can simultaneously prevent or limit the number of days on unnecessary plasma exchange and allow early initiation of appropriate and effective treatment.

Diagnosing vitamin B12 deficiency in patients presenting as pseudo-TMA requires thorough history taking, physical examination, and laboratory assessment. The PLASMIC score is a validated tool that can be used to screen for TTP as it correlates well with the ADAMTS13 level [6,7]. Patients with vitamin B12 deficiency-associated pseudo-TMA present with markers of hemolysis such as elevated LDH, as well as anemia and thrombocytopenia, and have a low vitamin B12 level, which is diagnostic [5,8]. Due to overlapping features with TTP, these patients can have an elevated PLASMIC score. Thus, the PLASMIC score cannot be reliably used to exclude this condition. A retrospective cohort study conducted in 2020 attempted to establish specific diagnostic criteria. It recommended reticulocytopenia, markedly elevated LDH, and an absence of significant thrombocytopenia as potential criteria. Of note, tear drop cells were also frequently noted on peripheral smears aside from schistocytes, and could thus be considered as an additional criterion [9].

## Review

Methodology

Literature Search

We followed the guidelines of Preferred Reporting Items for Systematic Reviews and Meta-Analyses (PRISMA) to conduct this review [10]. V Ganipisetti and S Maringanti independently searched PubMed, Embase, and Web of Science databases. The search strategies we used for each database are presented in Table 1.

**Table 1:** Summary of database search strategies.

Database	Search strategy
PubMed	We built the following search strategy using keywords and MeSH terms:(“vitamin b12 deficienc*”[Title/Abstract] OR “pernicious anemia”[Title/Abstract] OR “cobalamin deficiency”[Title/Abstract] OR “Vitamin B 12 Deficiency”[MeSH Terms] OR “anemia, pernicious”[MeSH Terms]) AND (“thrombotic microangiopath*”[Title/Abstract] OR “Thrombocytopenic purpura”[Title/Abstract] OR “Pseudo-TMA”[All Fields] OR “Thrombotic thrombocytopenic purpura”[Title/Abstract] OR “Pseudo thrombotic thrombocytopenic purpura”[Title/Abstract] OR “Pseudo TTP”[Title/Abstract] OR “Pseudo-thrombotic microangiopathy”[Title/Abstract] OR “MM-TMA”[All Fields] OR “MAHA”[All Fields] OR “microangiopathic hemolytic anemia”[Title/Abstract] OR “acquired amegakaryocytic thrombocytopenia”[Title/Abstract] OR “Thrombotic Microangiopathies”[MeSH Terms] OR “purpura, thrombotic thrombocytopenic”[MeSH Terms] OR “thrombotic thrombocytopenic purpura acquired”[Supplementary Concept] OR “anemia, hemolytic”[MeSH Terms])”. Additional filters were applied to include only case reports and a custom date range of 5 years from January 2018 to January 2023
Embase	‘thrombotic thrombocytopenic purpura’:ab,ti OR ‘thrombotic microangiopathy’:ab,ti OR ‘thrombocytopenic purpura-hemolytic uremic syndrome’:ab,ti OR ‘pseudo ttp’:ab,ti OR tma:ab,ti OR ttp:ab,ti AND ‘b12 deficiency’:ab,ti OR ‘pernicious anemia’:ab,ti OR ‘cyanocobalamin’:ab,ti
Web of Science	Database search using keywords

K Naha performed an additional manual search for relevant case reports and included 11 cases. K Naha and E Lingas sorted discrepancies. E Lingas compiled the final list for data extraction and analysis.

Inclusion and Exclusion Criteria

All case reports of adults with vitamin B12 deficiency presenting as thrombotic microangiopathy between January 2018 and January 2023 were included.

Exclusion criteria included pediatric cases, case reports without full text available, and articles not classified as case reports, such as review articles and letters to the editor.

Data Extraction and Quality Assessment

Full texts of all included case reports were reviewed. We used the Joanna Briggs Institute critical appraisal tool to assess included case reports [11]. This tool consists of eight clearly defined and concise questions with four possible choices (yes, no, unclear, and not applicable). The number of “yes” answers for each study across the nine checklist selection criteria was counted and used for the overall inclusion of the study to reduce the risk of bias. A total of 46 case reports were ultimately included. Baseline demographic data, symptomatology, laboratory investigations, information on the etiology of vitamin B12 deficiency, treatment strategies, and final outcomes were extracted and compiled.

Results

Search Selection Results

**Figure 1:**
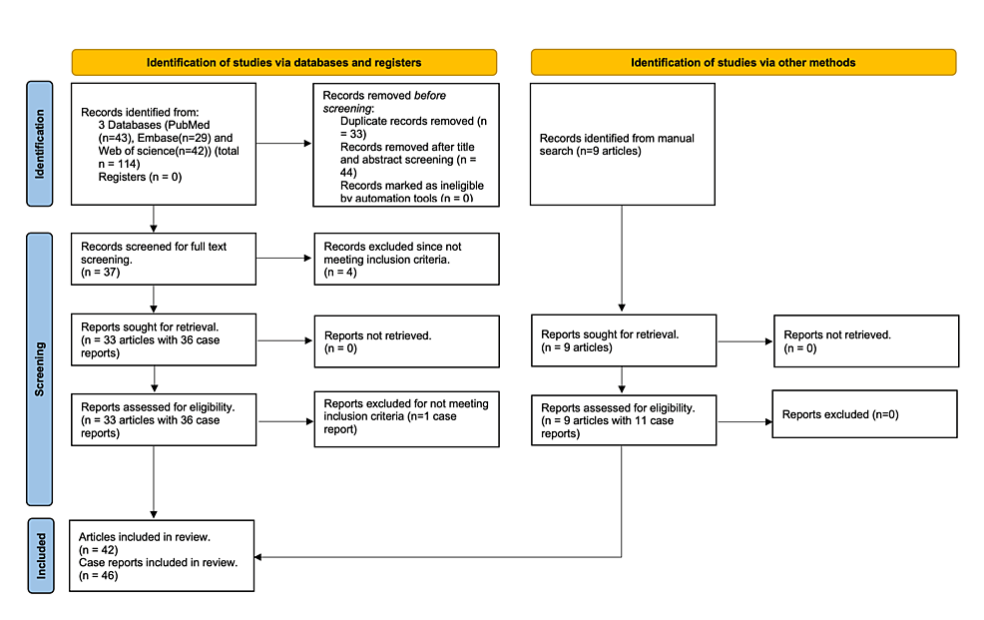
Preferred Reporting Items for Systematic Reviews and Meta-Analyses flowchart of the selection process.

A preliminary search of bibliographic databases yielded 114 case reports (43 from PubMed, 29 from Embase, 42 from Web of Science) with potential relevance to the topic. A total of 33 articles were excluded due to duplication. Further, 44 articles were excluded based on the exclusion criteria and irrelevance after screening the titles and abstracts. Of the remaining 37 articles, one article was excluded as it was a letter to the editor, and three other articles were excluded as the clinical presentation was with hemolysis only without other features of TTP. From the remaining 33 articles, 35 cases were extracted as these included two case series each describing two cases by Ceuleers et al. [12] and Jahangiri et al. [13]. An additional nine articles that included 11 case reports were identified by manual search. Finally, 46 cases were included for data extraction and analysis. The detailed process of identifying included articles is illustrated in Figure 1.

The list of case reports included in our review and a summary of each case report are presented in Table 2.

**Table 2:** Summary of the clinical characteristics of included cases. DOE = dyspnea on exertion; PA = pernicious anemia; DM = diabetes mellitus; HTN = hypertension; HIV = human immunodeficiency virus; GERD = gastroesophageal reflux disease; BPH = benign prostatic hyperplasia; FFP = fresh frozen plasma; RBC = red blood cells; N/A = not available; *H. pylori*: *Helicobacter pylori*

Authors, year of publication	Comorbidities	Symptoms and duration	Etiology of vitamin B12 deficiency	Peripheral smear/bone marrow findings	Treatments given
Abdelkhaleq et al., 2021 [14]	None	Asthenia and DOE for 1 week	PA	Schistocytes, hypersegmented neutrophils, and anisocytosis	Vitamin B12
Abosheaishaa et al., 2022 [15]	Alcohol use and subsequent pneumonia	Generalized weakness, with duration not mentioned	PA	Schistocytes	Vitamin B12
Abuhelwa et al., 2021 [16]	DM, HTN, and obesity	Dizziness, syncope, fall, and DOE for 3 months	PA	Schistocytes and hypersegmented neutrophils	Vitamin B12
Bailey et al., 2019 [17]	None	Fatigue, lower extremity numbness for 1 month, and acute jaundice	PA	Schistocytes and hypersegmented neutrophils	Prednisone, plasmapheresis, and vitamin B12
Buess et al., 2020 [18]	Hepatitis C	Fatigue for 1 month	Nutritional deficiency	Schistocytes and hypersegmented neutrophils	Vitamin B12
Ceuleers et al., 2022 [12]	None	Progressive exertional dyspnea for a few weeks	PA	Schistocytes	Vitamin B12, plasmapheresis, methylprednisolone, and caplacizumab
Ceuleers B et al., 2022 [12]	Euthyroid multinodular goiter	Fatigue and progressive dyspnea on exertion for 1 week	Nutritional deficiency	Schistocytes	Vitamin B12 and folic acid
Chang et al., 2021 [19]	Celiac disease	Fatigue, dizziness, and DOE for 1 week	Celiac disease	Schistocytes and macrocytosis	Vitamin B12
Chen et al., 2020 [20]	Iron deficiency anemia and GERD	Fatigue, vomiting, anorexia for 3 weeks, and weight loss for 6 months	PA	Schistocytes and anisocytosis	Plasmapheresis, followed by vitamin B12
Cochran et al., 2019 [21]	None	DOE for 1 month and 20 lbs weight loss	PA	Macrocytosis, schistocytes, tear drops, and hypersegmented neutrophils	Packed RBCs/FFP, oral vitamin K, and vitamin B12
Elharram et al., 2018 [22]	None	Fatigue and nausea for 2 weeks	PA	Macroovalocytes, hypersegmented neutrophils, and schistocytes	Vitamin B12
Elhiday et al., 2022 [23]	None	Fatigue and dyspepsia for 1 month	Nutritional deficiency	Hypersegmented neutrophils	Vitamin B12 and packed RBCs
Fahmawi et al., 2019 [24]	N/A	Fatigue and DOE for 2 weeks	PA	Schistocytes and nucleated RBCs	Vitamin B12 and plasmapheresis
Ganipisetti et al., 2023 [3]	Concomitant vitamin B1 deficiency	Weakness and confusion	Nutritional deficiency	N/A	Vitamin B12 and plasmapheresis
Gupta et al., 2022 [25]	N/A	Fatigue and fever for 1 week	Nutritional deficiency	Schistocytes	Vitamin B12, steroids, plasmapheresis, and rituximab
Harada et al., 2018 [26]	Gastric cancer and DM	Cognitive decline, purpura, and headache for 6 months	Gastrectomy	Schistocytes and hypersegmented neutrophils	Vitamin B12 and plasmapheresis
Hassouneh et al., 2021 [27]	Alcohol use	Fatigue, DOE, and loss of smell	PA	Hypersegmented neutrophils	Vitamin B12
Hussain et al, 2020 [28]	DM, iron deficiency anemia, and obesity	Fatigue and paresthesia for 6 weeks	Metformin use	Anisocytosis and schistocytes	Vitamin B12
Jahangiri et al., 2021 [13]	HTN, HLD, DM, BPH, and alcohol	DOE and fatigue for 1 week	Nutritional (alcohol)	Schistocytes	Vitamin B12
Jahangiri et al., 2021 [13]	Obesity and BPH	DOE, chest pain, and night sweats for 1 month	Unknown	Schistocytes	Vitamin B12
Jones et al., 2021 [29]	None	Fatigue	PA	Schistocytes	Vitamin B12
Jones et al., 2021 [29]	N/A	DOE for 2 weeks	Unknown	Schistocytes and macrocytosis	Vitamin B12 and plasmapheresis
Jones et al., 2021 [29]	Alcohol use	Fatigue and diarrhea	PA	Schistocytes	Vitamin B12
Kandel et al., 2017 [30]	N/A	Confusion for 2 days	PA	Schistocytes	Transfusion and vitamin B12
Katiyar et al., 2019 [31]	Stroke and deep vein thrombosis	Confusion for 1 month	PA	Schistocytes	Vitamin B12
Koontz et al., 2020 [32]	Graves’ disease	Intermittent diarrhea, bleeding gums, altered taste, and 30 lbs weight loss	Unknown	Hypersegmented neutrophils	Vitamin B12
Koubaissi et al., 2019 [33]	Hashimoto	Fatigue, weight loss for 1 month, dizziness, and DOE for 3 days	PA	Reactive cells and no schistocytes	Vitamin B12
Landry et al., 2021 [34]	Unknown	Fatigue, DOE, and weight loss	PA	Schistocytes	Vitamin B12
Lee et al., 2020 [35]	Diffuse large B-cell lymphoma	Fatigue, headache, and left upper quadrant pain	Suspected H. pylori infection	Hypersegmented neutrophils	Vitamin B12 and steroids
Li et al., 2021 [36]	Vitiligo	Confusion, vomiting, weight loss, paresthesia, and memory issues	PA	Schistocytes, hypersegmented neutrophils	Vitamin B12
Malhotra et al., 2019 [37]	Fungemia and brain bleed	Fall and DOE	PA	Schistocytes	Vitamin B12
Morrissey et al., 2022 [38]	Alcohol use	Chest pain, fatigue, DOE, and easy bleeding for 3 weeks	PA	Schistocytes	Plasmapheresis and vitamin B12
Nasrollahi et al., 2022 [39]	HTN	Left facial weakness and slurred speech for 6 weeks	Unknown	Schistocytes	Plasmapheresis, steroids, and vitamin B12
Navarro et al., 2018 [40]	Depression and learning disability	Nausea, abdominal pain, and fatigue	Homo-cystinuria and methylmalonic acidemia	Schistocytes	Plasmapheresis, eculizumab, and vitamin B12
Osman et al., 2021 [41]	HTN, hypothyroidism, and a history of bowel resection	Asymptomatic	Possible bowel resection related	Schistocytes	Vitamin B12
Perreira et al., 2021 [42]	None	Fatigue and anorexia for 3 months	PA	Schistocytes	Transfusion and vitamin B12
Pieralli et al., 2021 [43]	DM	DOE	Autoimmune gastritis	Schistocytes	Packed RBCs, vitamin B12, and plasmapheresis
Rao et al., 2020 [44]	N/A	DOE, anorexia, and fatigue for 2 weeks	PA	Schistocytes	Vitamin B12
Ricci et al., 2020 [45]	HIV	Headache, nausea, vomiting, and agitation	HIV-associated loss of haptocorrin	Large platelets	Plasmapheresis, vitamin B12, and rituximab
Sasi et al., 2020 [46]	N/A	Dizziness and fatigue for 3 days	PA	Schistocytes and hypersegmented neutrophils	Vitamin B12
Shigeta et al., 2021 [47]	HTN, stroke, and asthma	General weakness, anorexia, and coma	Unknown	Schistocytes	Plasmapheresis and vitamin B12
Sochat et al., 2019 [48]	Hepatitis C	Dizziness and weakness for several weeks	PA	Schistocytes	Plasmapheresis, steroids, and vitamin B12
Vanoli et al., 2018 [49]	Polysubstance abuse	Fatigue, myalgia, paresthesia, recurring headache, abdominal pain, and left ear tinnitus for 2 months	PA	Schistocytes	Vitamin B12
Wathieu et al., 2021 [50]	Osteoarthritis	Fatigue and dizziness for 3 weeks	PA	Schistocytes, hypersegmented neutrophils	Vitamin B12 and transfusion
Woodford et al., 2021 [51]	None	Chest pain and fatigue	Atrophic gastritis	Schistocytes and giant platelets	Steroids and vitamin B12
Yellala et al., 2018 [52]	Jehovah witness	Recurrent presyncope, fatigue, DOE, and weight loss of 30 lbs over 6 months	PA	Schistocytes and macro-ovalocytes	Vitamin B12

Demographics

The mean age of the included patients was 53.7 years (range = 17-90 years). More than half of the patients were male (63%), and 37% were female patients.

Comorbidities

A wide range of comorbidities were reported. Autoimmune comorbidities were reported in a few cases. Koontz et al. [32] reported a case with concomitant Graves’ disease, Koubaissi et al. [33] reported Hashimoto’s disease, Li et al. [36] reported vitiligo, and Chang et al. [19] reported a case of celiac disease. A few patients had a history of gastrectomy and alcoholism as comorbidities. Harada et al. [26] reported a history of gastric cancer, and Lee et al. [35] reported a history of lymphoma.

Symptomatology

Symptoms of this condition were mostly non-specific, such as fatigue, lightheadedness or dizziness, exertional dyspnea, and weakness. Some patients presented with neurological abnormalities, such as memory or cognitive issues, altered mental status, and paresthesias. Symptom onset could be days, weeks, or even a few months before presentation and diagnosis.

Laboratory Parameters

All included patients had low vitamin B12 levels, ranging from undetectable to 159 µ/L, with a mean level of 86.6 µ/L. Many patients presented with macrocytic anemia, with mean mean corpuscular volume (MCV) and hemoglobin values of 109.3 fL and 5 g/dL, respectively. Another notable laboratory abnormality was thrombocytopenia. Although the lowest platelet level observed was 8,000 cells/mm^3^, the mean platelet count at presentation was 77,400 cells/mm^3^. Elevated LDH was a common finding, with values ranging from 197 U/L to 9,894 U/L and a mean value of 3,165 U/L. Patients were also frequently found to have low haptoglobin. This combination of elevated LDH and depressed haptoglobin level is classically seen with hemolysis [53,54]. In vitamin B12 deficiency, however, these findings reflect intramedullary hemolysis due to ineffective erythropoiesis. The mean PLASMIC score was 5, with a maximum PLASMIC score of 6. Seven patients had a score of 6. Abnormal coagulation parameters, such as an elevated international normalized ratio, were reported in a few patients. Other reported abnormalities included elevated homocysteine levels and positive direct Coomb’s test in a few cases. The significance of these findings is unclear. A few included cases did not report PLASMIC scores, and we manually calculated values for them by reading the text and using available lab information. Reticulocyte index values were also not reported in a few case reports, and we did our final analysis on this using reported reticulocyte indexes only. The mean, median, maximum, and minimum values of relevant laboratory investigations were calculated and are presented in Table 3.

**Table 3:** Distribution of the demographic and laboratory markers of patients with cobalamin-deficient thrombotic microangiopathy. LDH = lactate dehydrogenase; WBC = white blood cell count; Hb = hemoglobin; MCV = mean corpuscular volume; NA = not applicable

	Mean	Median	Minimum, maximum	Normal range
Age (years)	53.7	59.5	17, 90	NA
LDH (U/L)	3,165	2,914	225, 9,894	140–280
Reticulocyte index (%)	0.9	0.6	0.005, 2.7	0.5–2.5
WBC (1,000/µL)	4.1	3.5	0.8, 11.4	4.5–11
Hb (g/dL)	5	4.9	1.8, 8.9	13.5–15
MCV (fL)	109.3	108.9	83.7, 134.3	80–100
Platelets (1,000/µL)	77.4	64	8, 222	150–450
Vitamin B12 level (pg/mL)	86.6	66	Undetectable, 159	200–900
PLASMIC score	5	5	4, 6	NA

Etiology

In our review, pernicious anemia was the most commonly identified etiology (56.5 %) for cobalamin-deficient thrombotic microangiopathy (26 out of 46 cases). Of the 46 case reports, six mentioned nutritional deficiency as an etiology. Of note, the case reported by Ganipisetti et al. also had concomitant thiamine deficiency [3], while the case reported by Ceuleers et al. had concomitant folate deficiency [12]. Hussain et al. reported metformin use as the etiology [28], and Ricci et al. reported human immunodeficiency virus-associated loss of haptocorrin as the etiology [45]. Chang et al. reported celiac disease as a possible etiology [19]. One case each of autoimmune gastritis, *Helicobacter pylori*, and atrophic gastritis etiologies was reported [35,43,51]. Other rare etiologies included gastric resection [26] and bowel resection [41]. Five cases did not mention any specific etiology.

Treatment

All 46 cases received vitamin B12 treatments with improvement in clinical condition. Fifteen patients (32.6%) also received plasma exchange (PLEX). The patients reported by Ricci et al. and Gupta et al. also had concomitant TTP and received rituximab [25,45]. Eight patients received steroids, one patient received eculizumab [40], and one patient received caplacizumab [12].

Discussion

Our review of cobalamin-deficient thrombotic microangiopathy cases revealed some key features that can help distinguish this disorder from TTP. The presence of risk factors for vitamin B12 deficiency, such as a strict vegetarian or vegan diet or poor nutrition, alcoholism, history of autoimmune diseases, gastrectomy, or gastric bypass, in a patient presenting with a TTP-like clinical picture should raise suspicion for cobalamin-deficient thrombotic microangiopathy. However, the absence of such factors cannot be used to rule out cobalamin deficiency, as the majority of patients in our review had undiagnosed pernicious anemia. Importantly, our review reinforced the clinical similarities between these two conditions, showing that symptomatology cannot be relied upon to distinguish between them.

Although the patients included in this review had laboratory findings of elevated LDH, low haptoglobin, and elevated indirect bilirubin similar to TTP, the degree of elevation of LDH was strikingly high compared to TTP. Our study showed a mean LDH of 3,165 U/L, whereas reviews of TTP have consistently shown elevated median LDH in the range of 1,107 to 1,750 U/L [55,56]. Previously published systematic reviews of cobalamin-deficient thrombotic microangiopathy also showed similar findings of a high median LDH [5].

Another distinctive feature in these patients was the presence of relatively modest thrombocytopenia with a median of 64,000/µL, and a mean of 77,500/µL, compared to the severe thrombocytopenia seen with TTP [55,57]. This finding likely reflects the underlying mechanism of inadequate production in contrast to the widespread thrombi formation leading to platelet consumption in TTP. Most patients in this review also presented with leukopenia with a median of 3.5 k//µL, often seen in severe vitamin B12 deficiency [58], but it is not a characteristic feature of TTP.

Of note, peripheral blood smears in most patients presented in this review demonstrated schistocytes, a hallmark of TTP. Taken along with biochemical features of hemolysis, this means that most of these cases fulfilled the criteria for microangiopathic hemolytic anemia (MAHA). The presence of hypersegmented neutrophils in peripheral smear should raise the suspicion of vitamin B12 deficiency.

The anemia observed in TTP results from intravascular hemolysis from MAHA and is associated with compensatory reticulocytosis due to bone marrow response to anemia. In contrast, even though cyanocobalamin deficiency can be associated with some features of hemolysis due to ineffective erythropoiesis, it is not associated with reticulocytosis and, indeed, usually presents with a hypoproliferative picture due to compromised marrow function. This phenomenon was reflected in the low reticulocyte index with a mean value of 0.9% seen in our review, which is consistent with a previous review [5].

Measurement of ADAMTS13 activity is the confirmatory test for the diagnosis of TTP [59], but it is often a send-out test and usually carries a slow turn-around time, which makes it impractical as a clinical decision-making tool when deciding whether to initiate PLEX. Bendapudi et al. [6] formulated a clinical scoring system called the PLASMIC score, which, when calculated, provides a low (4 and below), intermediate (5), or high (6 and above) probability of TTP. This allows rapid initiation of life-saving treatment for TTP while the ADAMTS13 level is awaited.

The median PLASMIC score for the patients in our review was 5, which falls under the intermediate category and necessitates initiation of treatment with plasmapheresis. In our review, we discovered that 15 patients received plasmapheresis, of whom five patients received concomitant systemic steroids. ADAMTS13 level was normal in all but two patients where TTP co-existed with vitamin B12 deficiency [25,45]. Based on these findings, it appears that the PLASMIC score cannot be used to distinguish between these two disorders reliably. Instead, we propose a modified PLASMIC score incorporating the reticulocyte index into the calculation to overcome this shortcoming. In a previous systematic review by Tran et al., the maximum reticulocyte production index (RPI) in the cases reviewed was 1.57% [5], and, in our review, the maximum reported value was 2.7% (Gupta et al.), where the patient also had concomitant TTP [25]. Usually, >3% RPI is considered a normal response to anemia [5]. This modified score would award -1 points for a reticulocyte index of less than 3% and 0 points for over 3%. This would lower the score to 4 in most of our patients, reclassifying them as low-risk and saving them from unnecessary PLEX and immunosuppressive therapy. Moreover, even with this modification, the two patients with concurrent TTP and vitamin B12 deficiency would have still scored 5 and thus received appropriate treatment. However, due to limited information available so far, further larger-scale studies are needed to validate this modified PLASMIC score, specifically by applying it to the registry of TTP patients and comparing the sensitivity and specificity of this score against the original PLASMIC score.

All patients included in our review responded to vitamin B12 supplementation with clinical improvement. Some patients received plasmapheresis which was later stopped after ruling out TTP. A few patients received steroids as well with no change in their clinical status [20,43,47,48].

Strengths and limitations

This systematic review has several advantages. To our knowledge, it is the only systematic review encompassing cases of cobalamin-deficient microangiopathy since 2018. The relevance of this study is underscored by its potential to contribute to the ongoing conversation about rising healthcare costs in the United States. Furthermore, the findings of this study highlight the pressing need to establish updated diagnostic guidelines to minimize unnecessary treatments such as PLEX and immunosuppression in this patient population.

The primary limitation of this study is its retrospective nature. Being sourced exclusively from published case reports, it is subject to bias. Consequently, the findings of this study may not precisely represent diagnostic thresholds. Many real-world cases may go undiagnosed and unreported and thus absent from the published literature, introducing an element of bias. We also had to manually calculate the PLASMIC score for a few case reports based on the information available in the case reports, as the exact score was not reported in the full text of the articles.

## Conclusions

Our systematic review adds to the medical literature on cobalamin-deficient thrombotic microangiopathy. Although uncommon, this condition can lead to diagnostic and clinical confusion because of several similarities that it shares with TTP. Our review supports the contention that patients with features of hemolytic anemia should be evaluated for vitamin B12 deficiency and that low vitamin B12 levels in such cases should be promptly managed by appropriate supplementation because of the high likelihood of reversing the pathologic process. Patients with cobalamin-deficient thrombotic microangiopathy tend to have higher LDH and platelet levels than those seen with true TTP and a lower reticulocyte index indicating a hypoproliferative bone marrow. We also found several case reports with normal MCV, indicating the importance of testing for vitamin B12 deficiency in these patients regardless of the MCV. We suggest incorporating the reticulocyte index into the PLASMIC score and validating it on TTP patients to increase its specificity without compromising its sensitivity. Future studies are needed to validate the diagnostic value of these laboratory abnormalities in distinguishing cobalamin-deficient thrombotic microangiopathy from TTP.

## References

[1] Noël N, Maigné G, Tertian G (2013). Hemolysis and schistocytosis in the emergency department: consider pseudothrombotic microangiopathy related to vitamin B12 deficiency. QJM.

[2] Stanley M, Killeen RB, Michalski JM (2024). Thrombotic Thrombocytopenic Purpura. https://www.ncbi.nlm.nih.gov/books/NBK430721/.

[3] Ganipisetti VM, Bollimunta P, Tun NN, Kanugula A, Anil V, Athavale A, Maringanti BS (2023). Concomitant vitamin B1 and vitamin B12 deficiency mimicking thrombotic thrombocytopenic purpura. Cureus.

[4] Tun AM, Myint ZW, Hernandez CR (2017). Vitamin B12 deficiency-related pseudo-thrombotic microangiopathy might be misdiagnosed and treated with plasma product therapy: review of the literature and analysis of the reported cases. Blood.

[5] Tran PN, Tran MH (2018). Cobalamin deficiency presenting with thrombotic microangiopathy (TMA) features: a systematic review. Transfus Apher Sci.

[6] Bendapudi PK, Hurwitz S, Fry A (2017). Derivation and external validation of the PLASMIC score for rapid assessment of adults with thrombotic microangiopathies: a cohort study. Lancet Haematol.

[7] Jamme M, Rondeau E (2017). The PLASMIC score for thrombotic thrombocytopenic purpura. Lancet Haematol.

[8] Walter K, Vaughn J, Martin D (2015). Therapeutic dilemma in the management of a patient with the clinical picture of TTP and severe B12 deficiency. BMC Hematol.

[9] Koshy AG, Freed JA (2020). Clinical features of vitamin B12 deficiency mimicking thrombotic microangiopathy. Br J Haematol.

[10] Moher D, Liberati A, Tetzlaff J, Altman DG (2009). Preferred reporting items for systematic reviews and meta-analyses: the PRISMA statement. BMJ.

[11] Munn Z, Moola S, Lisy K, Riitano D, Tufanaru C (2015). Methodological guidance for systematic reviews of observational epidemiological studies reporting prevalence and cumulative incidence data. Int J Evid Based Healthc.

[12] Ceuleers B, Stappers S, Lemmens J, Rutsaert L (2022). Cobalamin and folic acid deficiencies presenting with features of a thrombotic microangiopathy: a case series. Acta Clin Belg.

[13] Jahangiri P, Hicks R, Batth PK, Haas CJ (2021). Fooled by the fragments: vitamin B12 deficiency masquerading as thrombotic thrombocytopenic purpura. J Community Hosp Intern Med Perspect.

[14] Abdelkhaleq MA, Amal CH, Rime MO (2021). Don’t worry it’s just a vitamin B12 deficiency. Austin J Clin Case Rep.

[15] Abosheaishaa H, Nassar M, Ghallab M, Abdelwahed M, Ali A, Ibrahim B, Kimball E (2022). Pernicious anemia and vitamin B12 deficiency presenting as pseudothrombotic microangiopathy and developing secondary thrombocytopenia after treatment: a case report. Cureus.

[16] Abuhelwa Z, Khan T, Daas R, Ghazaleh S, Assaly R (2021). Life-threatening pseudo-thrombotic microangiopathy caused by severe vitamin B12 deficiency. Am J Med Case Rep.

[17] Bailey M, Maestas T, Betancourt R, Mikhael D, Babiker HM (2019). A rare cause of thrombotic thrombocytopenia purpura- (TTP-) like syndrome, vitamin B12 deficiency: interpretation of significant pathological findings. Case Rep Hematol.

[18] Buess CS, Germann AM, Maloney EP, Mohammed AB (2020). Vitamin B12 deficiency with pseudothrombotic microangiopathy and thrombotic thrombocytopenic purpura: similarities and differences. Kans J Med.

[19] Simone MC, Mercia G, Michael H (2021). Vitamin B12 malabsorption and pseudo- thrombotic microangiopathy in an adolescent. Thrombosis Update.

[20] Chen P, Ramachandran P, Josan K, Wang JC (2020). Pancytopenia and TTP-like picture secondary to pernicious anaemia. BMJ Case Rep.

[21] Cochran MK TA, Martinez E (2019). A pernicious mimicker: B12 deficiency presenting as splenomegaly, pancytopenia and hemolytic anemia. J Gen Intern Med.

[22] Elharram M, Sapon-Cousineau V, Tagalakis V (2018). Hemolytic anemia in a 26-year-old woman with vomiting and fatigue. CMAJ.

[23] Elhiday H, Musa M, Hussaini SA, Al-Warqi A, Alfitori G (2022). An unusual presentation of vitamin B12 deficiency associated with massive splenomegaly, hemolytic anemia, and pancytopenia: a case report. Cureus.

[24] Fahmawi Y, Campos Y, Khushman M (2019). Vitamin B12 deficiency presenting as pseudo-thrombotic microangiopathy: a case report and literature review. Clin Pharmacol.

[25] Gupta P, Ahmed S, Rout NK (2022). Co-existence of thrombotic thrombocytopenic purpura and megaloblastic anaemia: a case-based review. Mediterr J Rheumatol.

[26] Harada Y, Komori I, Morinaga K, Shimizu T (2018). Microangiopathic haemolytic anaemia with thrombocytopenia induced by vitamin B12 deficiency long term after gastrectomy. BMJ Case Rep.

[27] Hassouneh R, Shen S, Lee O, Hart RA, Rhea LP, Fadden P (2021). Severe vitamin B12 deficiency mimicking microangiopathic hemolytic anemia. J Hematol.

[28] Hussain SA, Ammad Ud Din M, Boppana LK, Kapoor A, Jamshed S (2020). Suspected metformin-induced cobalamin deficiency mimicking thrombotic thrombocytopenic purpura. Cureus.

[29] Jones C, Kumar R (2021). Vitamin B-12 deficiency and pseudo thrombotic microangiopathy: a case series. Thromb Haemost.

[30] Kandel S, Budhathoki N, Pandey S (2017). Pseudo-thrombotic thrombocytopenic purpura presenting as multi-organ dysfunction syndrome: a rare complication of pernicious anemia. SAGE Open Med Case Rep.

[31] Katiyar V, Qian E, Vohra I, Sleiman J, Rubinstein P (2019). Pernicious anemia presenting with pseudo thrombotic microangiopathy and falsely elevated B 12 levels. J Hematol.

[32] (2020). Abstracts from the 2020 Annual Meeting of the Society of General Internal Medicine. J Gen Intern Med.

[33] Koubaissi SA, Degheili JA (2019). Severe cobalamin deficiency disguised as schistocytes: a case report. Am J Case Rep.

[34] Landry I, Chowdhury T, Hussein S, Thomas L (2021). Life-threatening microangiopathy or vitamin deficiency: a case report of the clinical manifestations of pseudo-thrombotic microangiopathic anemia. Cureus.

[35] Lee KT, Teoh CS, Chew TK, Goh AS (2020). Microangiopathic haemolytic anaemia and thrombocytopenia due to combined vitamin B12 and folate deficiency masquerading as thrombotic thrombocytopenic purpura. J R Coll Physicians Edinb.

[36] (2021). Abstracts from the 2021 Annual Meeting of the Society of General Internal Medicine. J Gen Intern Med.

[37] Malhotra G, Kaur J, Patrawalla A, Berman A (2019). Pernicious anemia presenting with microangiopathy and multiorgan failure. Chest.

[38] Morrissey D, Sun Y, Koilpillai S, Kropf J, Carlan SJ (2022). Pseudo-thrombotic microangiopathy secondary to vitamin B12 deficiency. Case Rep Med.

[39] Nasrollahi F, Eilbert W (2022). Thrombotic microangiopathy presenting with stroke-like symptoms. Am J Emerg Med.

[40] Navarro D, Azevedo A, Sequeira S (2018). Atypical adult-onset methylmalonic acidemia and homocystinuria presenting as hemolytic uremic syndrome. CEN Case Rep.

[41] Osman H, Alwasaidi TA, Al-Hebshi A, Almutairi N, Eltabbakh H (2021). Vitamin B12 deficiency presenting with microangiopathic hemolytic anemia. Cureus.

[42] Pereira Fontes C, Fonseca S (2021). Pseudothrombotic microangiopathy as a rare presentation of cobalamin deficiency. Cureus.

[43] Pieralli F, Milia A, Fruttuoso S (2021). The doctor who stared at schistocytes: an intriguing case of suspected thrombotic microangiopathic anemia. Intern Emerg Med.

[44] Rao S, Colon Hidalgo D, Doria Medina Sanchez JA, Navarrete D, Berg S (2020). Et Tu, B12? Cobalamin deficiency masquerading as pseudo-thrombotic microangiopathy. Cureus.

[45] (2020). Special issue abstracts from the American Society for Apheresis Virtual Meeting, September 23-25, 2020. J Clin Apher.

[46] Sasi S, Yassin MA (2020). A rare case of acquired hemolytic anemia and pancytopenia secondary to pernicious anemia. Case Rep Oncol.

[47] Shigeta T, Sasaki Y, Maeda T, Hanji E, Urita Y (2021). Pseudo-thrombotic microangiopathy caused by acquired cobalamin deficiency due to unintentional neglect. Intern Med.

[48] Sochat M, Hermelin D, Chakos D (2019). When the diagnosis is difficult to digest: severe vitamin B-12 deficiency secondary to pernicious anemia mimicking life-threatening thrombotic thrombocytopenic purpura. J Hematopathol.

[49] Vanoli J, Carrer A, Martorana R, Grassi G, Bombelli M (2018). Vitamin B(12) deficiency-induced pseudothrombotic microangiopathy without macrocytosis presenting with acute renal failure: a case report. J Med Case Rep.

[50] Wathieu H, Bateman KM (2021). A case of pseudothrombotic microangiopathy associated with pernicious anemia. J Gen Intern Med.

[51] Woodford AM, Chaudhry R, Conte GA, Gupta V, Anne M (2021). Chronic atrophic gastritis presenting as hemolytic anemia due to severe vitamin B12 deficiency. Case Rep Hematol.

[52] Yellala A, Patibandla NSK, Shah R (2018). Vitamin B12 deficiency masquerading as TTP-to treat or not treat?. J Gen Intern Med.

[53] Galen RS (1982). Application of the predictive value model in the analysis of test effectiveness. Clin Lab Med.

[54] Marchand A, Galen RS, Van Lente F (1980). The predictive value of serum haptoglobin in hemolytic disease. JAMA.

[55] Page EE, Kremer Hovinga JA, Terrell DR, Vesely SK, George JN (2017). Thrombotic thrombocytopenic purpura: diagnostic criteria, clinical features, and long-term outcomes from 1995 through 2015. Blood Adv.

[56] Chiasakul T, Cuker A (2018). Clinical and laboratory diagnosis of TTP: an integrated approach. Hematology Am Soc Hematol Educ Program.

[57] Mariotte E, Azoulay E, Galicier L (2016). Epidemiology and pathophysiology of adulthood-onset thrombotic microangiopathy with severe ADAMTS13 deficiency (thrombotic thrombocytopenic purpura): a cross-sectional analysis of the French national registry for thrombotic microangiopathy. Lancet Haematol.

[58] Oh R, Brown DL (2003). Vitamin B12 deficiency. Am Fam Physician.

[59] Zheng XL (2015). ADAMTS13 and von Willebrand factor in thrombotic thrombocytopenic purpura. Annu Rev Med.

